# Cryptochrome and Period Proteins Are Regulated by the CLOCK/BMAL1 Gene: Crosstalk between the PPARs/RXRα-Regulated and CLOCK/BMAL1-Regulated Systems

**DOI:** 10.1155/2008/348610

**Published:** 2008-02-19

**Authors:** Koh-ichi Nakamura, Ikuo Inoue, Seiichiro Takahashi, Tsugikazu Komoda, Shigehiro Katayama

**Affiliations:** ^1^Department of Biochemistry, Saitama Medical University, 38 Morohongo, Moroyama, Iruma-gun, Saitama 350-0495, Japan; ^2^Department of Diabetes and Endocrinology, Saitama Medical University, 38 Morohongo, Moroyama, Iruma-gun, Saitama 350-0495, Japan

## Abstract

Feeding and the circadian system regulate lipid absorption and metabolism, and the expression of enzymes involved in lipid metabolism is believed to be directly controlled by the clock system. To investigate the interaction between the lipid metabolism system and the circadian system, we analyzed the effect of a CLOCK/BMAL1 heterodimer on the transcriptional regulation of PPAR-controlled genes through PPAR response elements (PPREs). Transcription of acyl-CoA oxidase, cellular retinol binding protein II (CRBPII), and 3-hydroxy-3-methylglutaryl coenzyme A (HMG-CoA) synthase was altered by CLOCK/BMAL1, and transcriptional activity via PPRE by PPARs/RXRα was enhanced by CLOCK/BMAL1 and/or by PPARs ligand/activators. We also found that CLOCK/BMAL1-mediated transcription of period (PER) and cryptochrome (CRY) was modulated by PPARα/RXRα. These results suggest that there may be crosstalk between the PPARs/RXRα-regulated system and the CLOCK/BMAL1-regulated system.

## 1. INTRODUCTION

Most living things, including humans, show a circadian rhythm in their activities. The molecular mechanism of this circadian rhythm has recently been clarified. The first discovery was identification of the period (PER) gene in *Drosophila* [1–3]. Later, the single minded (SIM) and aryl hydrocarbon receptor nuclear translocator (ARNT) isoforms were discovered in *Drosophila*, and ARNT was identified as a dioxin receptor and as a transcription factor. Thereafter, the three genes (*PER, ARNT, and SIM*) were shown to have identical domains, the so-called PAS domain [4]. In addition, these transcription factors contain the basic-helix-loop-helix (bHLH)-leucine zipper domains, and SIM and ARNT form heterodimers [5]. Another variant of these genes was named the CLOCK gene (*CLOCK*) by Takahashi et al. [6, 7]. Moreover, Ikeda et al. [8] cloned the brain and muscle Arnt-like protein 1 (BMAL1) gene (*BMAL1*) from brain and muscle tissue and demonstrated its homology to ARNT, including possession of a bHLH-PAS domain. Recent studies using the two-hybrid method have shown that BMAL1 forms a heterodimer with *CLOCK*. This CLOCK/BMAL1 heterodimer can bind to the mouse PER gene (*mPER*) promoter region, and act as a transcriptional regulator [9]. The base sequence $5^{\prime }$-CACGTG-$3^{\prime }$, which is located in the promoter region, has been named the E-box. There are three isoforms of the PER gene *(PER1,2,3)*. In addition, cryptochrome genes (*CRY1,2*) have been identified [10]. Interacting positive and negative transcriptional-translational feedback loops have been reported to drive circadian oscillations in both *Drosophila* and mammals. The best-characterized feedback loop is in the mouse and involves the regulation of three m*PER1-3* and two m*CRY1-2* genes [11]. It is now thought that transcription of m*PER* and m*CRY* is driven by accumulating CLOCK/BMAL1 heterodimers, which in turn bind to consensus E-box elements ($5^{\prime }$-CACGTG-$3^{\prime }$) [9].

The peroxisome proliferator-activated receptor (PPAR) is a member of the nuclear receptor superfamily [12]. Three types of PPARs have been described in rodents, humans, and amphibians: PPARα, PPARβ (also called PPARδ), and PPARγ. PPARα is expressed most abundantly in the liver [13] and is associated with β-oxidation in the mitochondria and peroxisomes. Although PPARβ/δ is expressed ubiquitously [14], its functional role remains to be clarified. Activation of PPARα decreases the fatty acid content of cells through β-oxidation of fatty acids, whereas PPARγ is a transcription factor expressed selectively in adipose tissue [15] and seems to be associated with adipocyte differentiation. Fenofibric acid is a synthetic ligand of PPARα, and the thiazolidinedione drugs such as troglitazone are synthetic ligands of PPARγ. No synthetic ligands of PPARβ/δ are so far used clinically.

Steroid hormones such as dexamethasone have been found to regulate these three PPARs. It is well known that circulating concentrations of some steroids exhibit circadian variability [16], and recent observations suggest that feeding cycles are capable of regulating peripheral clocks independent of light stimulation [17]. A recent study has also shown that the circadian expression of the PPAR gene (*PPAR*) is regulated by peripheral oscillators in a CLOCK-dependent manner [18]. Moreover, we have also shown that CLOCK and BMAL1 play an important role in lipid homeostasis by regulating the circadian activation of potential PPAR response element- (PPRE-) controlled target genes [19]. The partner of PPAR, RXR, interacts with the CLOCK protein in a ligand-dependent manner and inhibits CLOCK/BMAL1-dependent activation via an E-box element [20]. McNamara et al. also recently reported that the retinoid X receptor (RXR) and retinoic acid receptor (RAR) are capable of interacting with MOP4 and CLOCK. MOP4 is a transcription factor, and is also called NPAS2. MOP4 is a paralog of CLOCK [21, 22] that can dimerize with BMAL1 and appears to function in a clockwork mechanism in mouse forebrain. RXR has been shown to bind to PPARs, resulting in formation of the heterodimer, which then binds to the peroxisome proliferator response element (PPRE), thereby activating target genes including those coding for acyl-CoA oxidase (AOX), lipoprotein lipase, acyl-CoA synthase, cellular retinol binding protein II (CRBPII), and 3-hydroxy-3-methylglutaryl coenzyme A (HMG-CoA) synthase [23, 24].

In the present study, we used AOX, CRBPII, and HMG-CoA synthase promoter reporters to investigate whether their transcriptional activity is regulated by the CLOCK/BMAL1 heterodimer. We also investigated whether CLOCK/BMAL1 heterodimers are capable of modulating the expression of PPAR/RXR-regulated genes through the endogenous factors regulated by CLOCK/BMAL1.

## 2. MATERIALS AND METHODS

### 2.1. Reagents

Fenofibric acid was provided by Kaken Pharmaceutical Co. (Tokyo, Japan). We used 10 *μ*M fenofibric acid as a PPARα ligand in our experiments, a concentration that is below its reported EC_50_ value [25].

Troglitazone was generously provided by Sankyo Co. (Tokyo, Japan). The cytotoxicity of the compounds tested was assessed by the trypan blue dye exclusion method.

### 2.2. DNA sequencing

DNA sequencing was performed with an automated sequencer (ABI PRISM 310 Genetic Analyzer; Perkin Elmer, Foster City, Calif, USA). All DNA sequences were confirmed by reading both DNA strands.

### 2.3. Western blotting

An Amersham ECL kit was used to perform Western blotting to detect specific immunoreactivity. Briefly, heat-treated 15 *μ*g samples of CLOCK/BMAL1 were subjected to 10% sodium dodecylsulfate polyacrylamide-gel electrophoresis and transferred to nitrocellulose membranes (Millipore) by semidry blotting. Molecular weight was determined by using markers (RAINBOW Molecular Weight Markers, RPN800; Amersham Biosciences, Buckinghamshire, UK). Antibodies against the proteins coded by the human clock genes *PER*1, *PER*2, *CRY*1, and *CRY*2 were obtained from Santa Cruz Biotechnology (Calif, USA).

Membranes were treated overnight with TBS-Tween: 5% dry milk and incubated for 1 hour with 1000-fold diluted goat antibodies to human PER1, PER2, CRY1, and CRY2. After washing, the membranes were incubated with horseradish-peroxidase-conjugated rabbit antigoat monoclonal antibodies. Antigen detection was performed with a chemiluminescence detection system.

### 2.4. Human PPARα, PPARγ1, RXRα, CLOCK, and BMAL1 expression constructs

DNA fragments encoding full-length human PPARα (Genebank L-02932), PPARγ1 (Genebank L-40904), and RXRα (Genebank X-52773) cDNAs were amplified from skeletal muscle cDNA (no. 9514, Takara Shuzo Co., Ltd., Kyoto, Japan) or liver cDNA (no. 9505, Takara Shuzo Co., Ltd.) and ligated into pCR2.1 (Invitrogen). For PPARα, the 1407 bp *Sal* I/*Not* I fragment was subcloned into the *Sal* I/*Not* I site of pCI-neo (Promega), and the vector was named pCI-PPARα. PPARγ1 was subcloned into the *Sal* I/*Not* I site of the vector, and the vector was named pCI-PPARγ1. RXRα was also subcloned into the *Xho* I/*Not* I site of the same vector, and the vector was named pCI-RXRα.

Human full-length cDNAs for CLOCK and BMAL1 were generated by the method reported previously [26]. The cDNA fragments for CLOCK and BMAL1 were subcloned into the *Sal* I/*Not* I site of pcDNA3 (Invitrogen, Calif, USA), and the vectors were named pc-CLOCK and pc-BMAL1, respectively.

### 2.5. Luciferase reporter assay

Transient transfections for the reporter assay were performed with Tfx-50 reagent (Promega) by following the protocol recommended by the manufacturer. 293T human kidney cells (293T cells), CV-1 monkey kidney cells (CV-1 cells), NIH3T3 cells, and COS-1 cells were used because of their high transfection efficiency.

All luciferase reporter assays were performed using charcoal-dextran-treated medium (no. SH30068.03; HyClone, Utah, USA). When medium is treated with charcoal, bioactive factors, such as testosterone, thyroxine, triiodothyronine, vitamin A, 1,25-dihydroxy-vitamin D, and 1β7-estradiol, are adsorbed by the charcoal, and the concentration of all these hormones is greatly reduced (as stated in the manufacturer’s protocol). Since all these hormones act through nuclear receptors such as PPAR, all luciferase-reporter assays were performed using charcoal-dextran-treated medium.

We used AOX, CRBPII, and HMG-CoA synthase promoter regions [24] ligated into the *Kpn* I/*Nco* I sites of pGL3-Basic (Promega) upstream of the luciferase gene, and called these constructs pAOX-Luc, pCRBPII-Luc, and pHMG-Luc, respectively. Typically, genes (3 μg) were inserted into $1\times 10^{6}$ cells in 100-mm dishes for pAOX-Luc, pCRBPII-Luc, or pHMG-Luc, and for pCI-PPARα, pCI-PPARγ1, pCI-RXRα, pcDNA-CLOCK, or pcDNA-BMAL1. To measure transcription rates induced by PPARs/RXRα and CLOCK/BMAL1 via the E-box, we used the promoter region of m*PER1* cloned into the *Kpn* I/*Nco* I sites of pGL3-Basic upstream of the luciferase gene, a construct named pPER-Luc. There are three E-box elements ($5^{\prime }$-CACGTG-$3^{\prime }$) in the promoter region (about 2,000 base pairs) of m*PER1* [26].

pPPRE-Luc, pCRBPII-Luc, pHMG-Luc or pPER-Luc, together with pRL-TK, in the presence or absence of pCI-PPARα, pCI-PPARγ1, pCI-RXRα, pcDNA-CLOCK, or pcDNA-BMAL1 vectors, were cotransfected into cells which were grown in 24-well plates. The total amount of DNA transfected (0.6 μg) was normalized with a carrier DNA (pCI-neo or pcDNA3). After 24 hours, cells were stimulated with the PPARs ligand/activator for another 24 hours. Finally, luciferase activity for pAOX-Luc, pCRBPII-Luc, pHMG-Luc, or pPER-Luc was normalized to *Renilla* luciferase activity. All these activities were measured according to the manufacturer's instructions (Promega).

### 2.6. Transient transfection/cotransfection

pCI-PPARα (3 μg), pCI-PPARγ1 (3 μg), pCI-RXRα (3 μg), pcDNA-CLOCK (3 μg), or pcDNA-BMAL1 (3 μg) were transiently transfected into $1\times 10^{6}$ cells in 100 mm dishes by using Tfx-50 Reagent (Promega) according to the manufacturer’s instructions. After 24 hours, the cells were stimulated with a test compound for 24 hours and were then analyzed by Western blot assay.

### 2.7. Deletion models for the CRBPII-Luc plasmid (see Figure 1), pAOX-Luc plasmid, and pHMG-Luc plasmid

The CRBPII gene promoter ($5^{\prime }$-gtgtcccactctgctgtcac**AGG** **TCA**c**AGGTCA**c**AGGTCA**c**AGTTCA**ttttcctgtctctgtc-$3^{\prime }$, from −670 to +63) contains four ($5^{\prime }$-AG(G/T)TCA-$3^{\prime }$) repeat sequences [23]. We determined whether or not the action of CLOCK and BMAL1 on the CRBPII promoter was dependent on the number of AGGTCA sequences. Deletions were introduced into the CRBPII-Luc plasmid (from −670 to +63) by the PCR method or with the Exo/Mung Bean Deletion kit (Stratagene, Calif, USA) to generate the following reporter constructs: del-0-CRBPII-Luc ($5^{\prime }$-cctgtctctgtc-$3^{\prime }$, from −599 to +63), del-1-CRBPII-Luc ($5^{\prime }$-c**AGGTCA**ttttcctgtctctgtc-$3^{\prime }$, from −610 to +63), del-2-CRBPII-Luc ($5^{\prime }$-c**AGGTCA**c**AGGTCA** ttttcctgtctctgtc-$3^{\prime }$, from −617 to +63), del-3-CRBPII-Luc ($5^{\prime }$-c**AGGTCA**c**AGGTCA**c**AGGTCA**ttttcctgtctctgtc-$3^{\prime }$, from −624 to +63), and del-4-CRBPII-Luc ($5^{\prime }$-c **AGG** **TCA**c**AGGTCA**c**AGGTCA**c**AGGTCA**ttttcctgtctctgtc-$3^{\prime }$, from −631 to +63). The del-0-CRBPII-Luc plasmid contained no ($5^{\prime }$-AG(G/T)TCA-$3^{\prime }$) repeat sequences; the del-1-CRBPII-Luc plasmid contained one ($5^{\prime }$-AG(G/T)TCA-$3^{\prime }$) repeat sequence; the del-2-CRBPII-Luc plasmid contained two ($5^{\prime }$-AG(G/T)TCA-$3^{\prime }$) repeat sequences; the del-3-CRBPII-Luc plasmid contained three ($5^{\prime }$-AG(G/T)TCA-$3^{\prime }$) repeat sequences, and the del-4-CRBPII-Luc plasmid contained four ($5^{\prime }$-AG(G/T)TCA-$3^{\prime }$) repeat sequences.

Deletions were also introduced into the pAOX-Luc plasmid or pHMG-Luc plasmid to generate reporter constructs by the same methods as for the deletions of the CRBPII-Luc plasmid.

### 2.8. Statistical analysis

Four independent experiments were performed, and each experiment was performed in triplicate. Parametric data are expressed as the mean ± standard deviation (SD). Differences between groups were evaluated by Scheffé's F test. Differences were analyzed by repeated-measures ANOVA.

## 3. RESULTS AND DISCUSSION

To determine whether there is crosstalk between the PPRE-mediated transcription system and the E-box-mediated transcription system, we first measured transcriptional activation of the target genes by PPARs/RXRα and CLOCK/BMAL1 via PPRE. All the experiments were performed with human kidney 293T cells and CV-1 monkey kidney cells. Because introduction of pAOX-Luc and pCRBPII-Luc into human kidney 293T cells, and of pHMG-Luc into CV-1 monkey kidney cells, was particularly efficient, only these results are presented.

**Figure 2:**
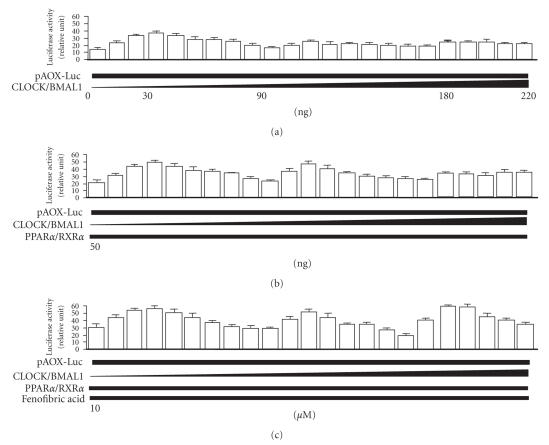
Dose-dependent alteration by the *CLOCK/BMAL1* gene (0–220 ng) of the transcriptional activity of PPARα/RXRα mediated through PPRE ($5^{\prime }$-AGGTCA-$3^{\prime }$) elements located in the promoter of acyl-CoA oxidase (AOX) in human kidney 293T cells in charcoal-treated medium. Alteration by the *CLOCK/BMAL1* gene alone (a), plus the *PPARα/RXRα* gene (50 ng) (b), and plus the PPARα ligand/activator fenofibric acid (10μM) (c) in activity of transcription by PPARα/RXRα through PPRE ($5^{\prime }$-AGGTCA-$3^{\prime }$) elements located in the promoter of AOX. Luciferase activity for pAOX-Luc was normalized to the luciferase activity of pRL-TK. Experiments were performed in triplicate, and four independent experiments were performed. All data are means ± SD.

The effect of *CLOCK/BMAL1* (see Figure 2) on transcriptional activity of *AOX* (pAOX-Luc) was determined by a reporter gene assay in 293T cells. PPARα/RXRα-mediated transcription of the AOX gene via PPRE in 293T cells was enhanced in charcoal-treated medium by cotransfection with the CLOCK/BMAL1 expression constructs (0∼30 ng). Unexpectedly, the enhanced PPARs/RXRα-mediated transcription of the AOX gene was significantly decreased by cotransfection of higher amounts (30∼90 ng) of the CLOCK/BMAL1 expression constructs in charcoal-treated medium (see Figure 2). When luciferase-reporter assays were performed using medium not treated with charcoal-dextran, the results were the exact opposite. Thus, different results are obtained depending on whether the sample is treated or is not treated with charcoal-dextran (data not shown). There are indications that serum stimulation can regulate the transcription of clock genes [27], and the levels of factors regulating such genes may be altered in culture media when samples are treated with charcoal-dextran. Shirai et al. [28] have reported that PPARα is involved in circadian clock control independently of the suprachiasmatic nucleus (SCN), which is the central clock in mammals. Therefore, it is possible that a PPAR ligand acts on the expression of CLOCK/BMAL1, and that consistent results depend on the complete removal of this ligand from the serum used.

The retinoid receptors RXR and RAR are capable of interacting with MOP4 and CLOCK [20]. The diminished PPARs/RXRα-mediated transcription caused by cotransfection of larger amounts of the CLOCK/BMAL1 heterodimer (30∼90 ng) may be attributable to binding between CLOCK and RXRα. However, binding between CLOCK and RXRα does not appear to occur after cotransfection of smaller amounts of the CLOCK/BMAL1 heterodimer (0∼30 ng), indicating that there may be an optimal ratio of *CLOCK/BMAL1* to *PPARα/RXRα*. The CLOCK, BMAL1, PPARα, and RXRα gene products form heterodimers via the bHLH domain to act as transcription factors. As mentioned above, McNamara et al. have reported that the partner RXRα gene product for PPARα can bind to CLOCK, resulting in decreased transcriptional activity of PPARα [20]. Previously, we have found that RXRα can bind to CLOCK and, further, that PPARα can bind to CLOCK. In addition, PPARα can bind to BMAL1, and RXRα can bind to CLOCK. The binding affinities of these interactions are different (Inoue I: Crosstalk between PPARα/RXRα gene and clock gene (BMAL1/CLOCK). 35th, Japan Atherosclerosis Society, Kyoto , September, 2003). The relative transcriptional activities of the various heterodimers of CLOCK, BMAL1, PPARα, and RXRα remain to be determined.

**Figure 3:**
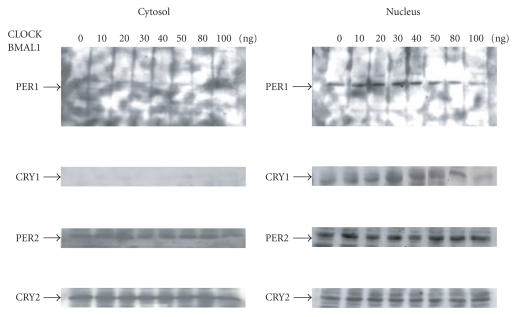
Western blotting analysis demonstrating changes in Period 1 (PER1), Period 2 (PER2), Cryptochrome 1 (CRY1), and Cryptochrome 2 (CRY2) protein levels in the cytosol and nucleus by the *CLOCK/BMAL1* gene (0–100 ng). Experiments were performed in triplicate, and four independent experiments were performed. A typical set of data is shown.

We performed a Western blot analysis of 293T cells transfected with various amounts of *CLOCK/BMAL1* (0∼100 ng). The increase in CRY1 and PER1 protein levels in the nucleus peaked at the 30 ng dose of *CLOCK/BMAL1* (see Figure 3). Transcription of the mPER and mCRY genes is now thought to be induced by accumulating CLOCK/BMAL1 heterodimers, which, in turn, bind to consensus E-box elements. A consensus E-box element is present in the promoter of the PER and CRY genes. That is, the CLOCK/BMAL1 heterodimer binds to the E-box, $5^{\prime }$-CACGTG-$3^{\prime }$, located in the promoter regions of the PER and CRY genes, such that the PER and CRY genes can be regulated by the CLOCK/BMAL1 heterodimer. The PER and CRY gene products have been shown to inhibit the binding of CLOCK/BMAL1 to consensus E-box elements of the PER and CRY genes, resulting in a reduction in the transcriptional activity of these genes [9, 29, 30]. From our results, DNA binding of the CLOCK/BMAL1 heterodimer can be regulated by the protein products of the PER and CRY genes, suggesting that the PER and CRY gene products can alter the transcriptional activity of the AOX, CRBPII, and HMG-CoA synthase genes via their PPRE. Further research is necessary to confirm this suggestion. Oishi et al. [18] have reported that CLOCK is involved in the transactivation of PPARα. There is the possibility that increased protein expression of PPARα caused by the CLOCK gene acts directly on the PPRE. The increases in endogenous PER1 and CRY1 protein levels induced by exogenous CLOCK/BMAL1 may decrease the PPRE-mediated transcriptional activity of AOX. Another report suggests that CRY is capable of acting directly on CLOCK/BMAL1, which would mean that CRY is capable of regulating the gene expression of CLOCK/BMAL1 [31]. Our results suggest that the promoter transcriptional activity of pCRBPII could be modulated by the CLOCK and BMAL1 genes within the dose range 0–100 ng. However, the change in pCRBPII promoter transcriptional activity induced by the CLOCK and BMAL1 genes also showed the same tendency over the dose range 100–220 ng (data not shown), suggesting a similar mechanism of action.

**Figure 4:**
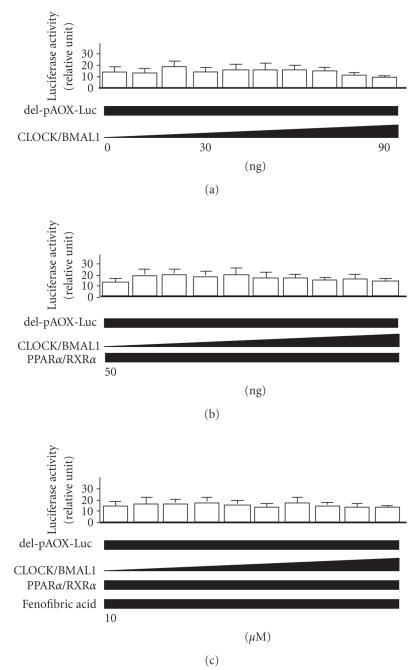
Dose-dependent changes in activity of PPARα/RXRα-mediated transcription via the acyl-CoA oxidase (AOX) deletion promoter by the *CLOCK/BMAL1* gene (0–90 ng) in human kidney 293T cells. Alterations in PPRE-mediated transcription of AOX by the *CLOCK/BMAL1* gene alone (a), plus the *PPARα/RXRα* gene (50 ng) (b), and plus the PPARα ligand/activator fenofibric acid (10 μM) (c). The luciferase activity of del-pAOX-Luc was normalized to the luciferase activity of pRL-TK. The del-AOX-Luc plasmid contained no repeat sequences ($5^{\prime }$-AGGTCA-$3^{\prime }$). Experiments were performed in triplicate, and four independent experiments were performed. All data are means ± SD.

The pAOX-Luc construct contains six ($5^{\prime }$-AGGTCA-$3^{\prime }$) repeat sequences, and when all six ($5^{\prime }$-AGGTCA-$3^{\prime }$) repeat sequences were deleted, no change in transcriptional activity of the AOX promoter was observed in 293T cells transfected with the same amounts of CLOCK/BMAL1 in charcoal-treated medium (see Figure 4).

**Figure 5:**
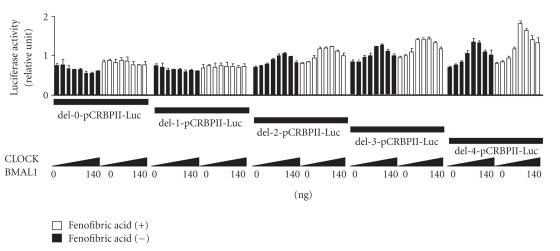
Dose-dependent changes in activity of the cellular retinol binding protein II (CRBPII) promoter in human kidney 293T cells induced by the *CLOCK/BMAL1* gene (0–140 ng) plus the *PPARα/RXRα* gene (50 ng). The del-0-CRBPII-Luc plasmid contained no repeat sequences ($5^{\prime }$-AG(G/T)TCA-$3^{\prime }$); the del-1-CRBPII-Luc plasmid contained one repeat sequence ($5^{\prime }$-AG(G/T)TCA-$3^{\prime }$); the del-2-CRBPII-Luc plasmid contained two repeat sequences ($5^{\prime }$-AG(G/T)TCA-$3^{\prime }$); the del-3-CRBPII-Luc plasmid contained three repeat sequences ($5^{\prime }$-AG(G/T)TCA-$3^{\prime }$), and the del-4-CRBPII-Luc plasmid contained four repeat sequences ($5^{\prime }$-AG(G/T)TCA-$3^{\prime }$). The luciferase activity of all pCRBPII-Luc plasmids was normalized to the luciferase activity of pRL-TK. Experiments were performed in triplicate, and four independent experiments were performed. All data are means ± SD.

As stated above, the CRBPII promoter also contains four ($5^{\prime }$-AG(G/T)TCA-$3^{\prime }$) repeat sequences, and when all four ($5^{\prime }$-AG(G/T)TCA-$3^{\prime }$) repeat sequences were deleted, no change in transcriptional activity of the CRBPII promoter was observed in 293T cells transfected with the same amounts of CLOCK/BMAL1 in charcoal-treated medium (see Figure 5). When the CRBPII promoter containing one repeat sequence ($5^{\prime }$-c**AGTTCA**t-$3^{\prime }$), two repeat sequences ($5^{\prime }$-c**AGGTCA**c**AGTTCA**t-$3^{\prime }$), or three repeat sequences ($5^{\prime }$-c**AGGTCA**c**AGGTCA**c**AGTTCA**t-$3^{\prime }$) was transfected, the change in transcriptional activity of the CRBPII promoter gradually increased in 293T cells transfected with the same amounts of CLOCK/BMAL1. When transfected with promoter containing four repeat sequences ($5^{\prime }$-c**AGGTCA**c**AGGTCA**c**AGGTCA**c**AGTTCA**t-$3^{\prime }$), the expected change in transcriptional activity of the CRBPII promoter was observed in 293T cells transfected with the same amounts of CLOCK/BMAL1 (see Figure 5).

**Figure 6:**
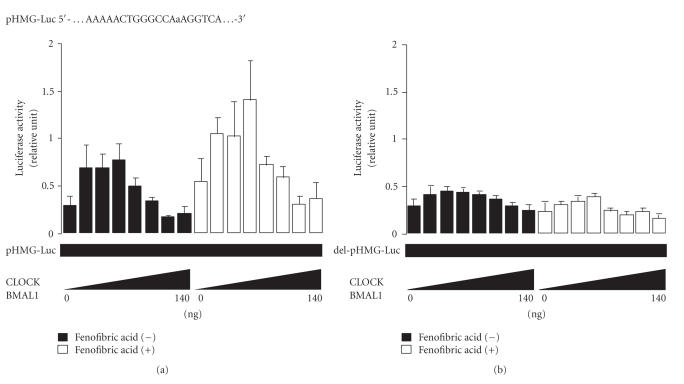
The effect of the CLOCK/BMAL1 heterodimer on PPARα target genes. Dose-dependent changes in activity of the 3-hydroxy-3-methylglutaryl coenzyme A (HMG-CoA) synthase promoter (a) induced by the *CLOCK/BMAL1* gene (0–140 ng) in the presence and absence of fenofibric acid (10 μM) in CV-1 monkey kidney cells plus the *PPARα/RXRα* gene (50 ng). When the promoter of the HMG-CoA synthase gene did not contain direct repeat sequences (del-pHMG-Luc), no change in transcriptional activity of the HMG-CoA synthase promoter was observed (b). The luciferase activity of pHMG-Luc was normalized to the luciferase activity of pRL-TK. Experiments were performed in triplicate, and four independent experiments were performed. All data are means ± SD.

A similar tendency was observed in CV-1 cells transfected with the promoter of the HMG-CoA synthase gene (see Figure 6(a)), which contains direct ($5^{\prime }$-AAAAACT**GGGCCA**a**AGGTCT**-$3^{\prime }$) repeat sequences [24]. When these direct ($5^{\prime }$-AAAAACT**GGGCCA**a**AGGTCT-$3^{\prime }$**) repeat sequences were deleted from the promoter of the HMG-CoA synthase gene, no change in transcriptional activity of the HMG-CoA synthase promoter was observed in CV-1 cells transfected with the same amounts of CLOCK/BMAL1 in charcoal-treated medium (see Figure 6(b)).

**Figure 7:**
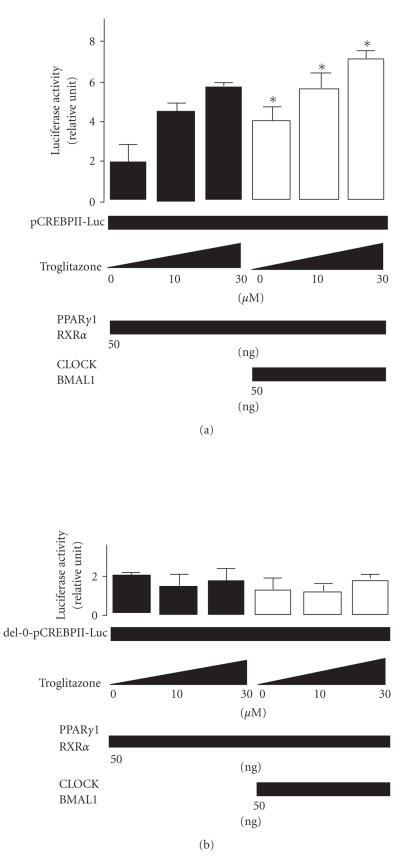
The effect of the CLOCK/BMAL1 heterodimer on PPARγ target genes. Changes in activity of PPARγ1/RXRα-mediated (50 ng) (a) transcription of the cellular retinol binding protein II (CRBPII) promoter by the *CLOCK/BMAL1* gene (50 ng) in human kidney 293T cells in the presence and absence of the PPARγ ligand/activator troglitazone (0–30μM). The del-0-CRBPII-Luc plasmid contained no repeat sequences ($5^{\prime }$-AG(G/T)TCA-$3^{\prime }$). The luciferase activity of pCRBP-Luc was normalized to the luciferase activity of pRL-TK. Experiments were performed in triplicate, and four independent experiments were performed. All data are means ± SD. *P < .05 versus cells not transfected with the *CLOCK/BMAL1* gene. Differences were analyzed by repeated-measures ANOVA.

A similar tendency toward changes in transcriptional activity of the CRBPII promoter (see Figures 7(a), 7(b)) was observed when the PPARγ ligand/activator troglitazone was added. Transcription of AOX, CRBPII, and HMG-CoA synthase was increased by CLOCK/BMAL1, and the transcriptional activity via the PPRE of PPARs/RXRα was enhanced by CLOCK/BMAL1 and/or PPARs ligand/activator. These results shown in Figures 6 and 7 clearly indicate that CLOCK/BMAL heterodimer acts as a positive functional regulator of PPARα and PPARγ1. However, it is not clear that whether CLOCK/BMAL heterodimer directly reacts with the promoter regions of target genes of PPARs, or bounds to the PPARs /RXRα proteins themselves.

**Figure 8:**
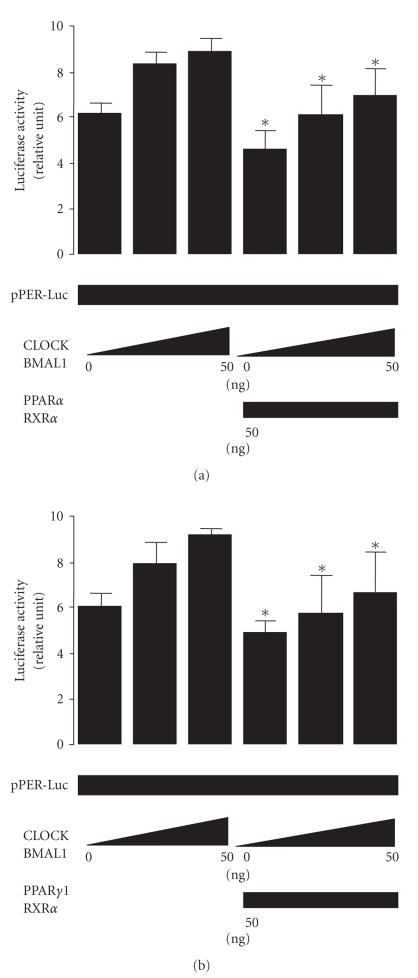
Inhibition of CLOCK/BMAL1 by *PPARα* and *PPARγ1.* The *PPARα/RXRα* (a) gene (50 ng) and the *PPARγ1/RXRα* (b) gene (50 ng) abolished activation of the *Period1* (*Per1*) promoter by the *CLOCK/BMAL1* gene (0–50 ng) in human kidney 293T cells in the presence of the PPARα ligand/activator fenofibric acid (10 μM) (a) and PPARγ ligand/activator troglitazone (10 μM) (b). The luciferase activity of pPER-Luc was normalized to the luciferase activity of pRL-TK. Experiments were performed in triplicate, and four independent experiments were performed. All data are means ± SD. *P < .05 versus cells not transfected with the *PPARα/RXRα* gene or *PPARγ1/RXRα* gene. Differences were analyzed by repeated-measures ANOVA.

Next, to determine whether the effect of *CLOCK/BMAL1* on transcription of mPER is modulated by PPARα/RXRα or PPARγ1/RXRα, we investigated the effect of the *CLOCK/BMAL1* gene on transcription of the *PER* gene in 239T cells. Transcription of the mPER1 gene was altered by co-transfection with the CLOCK/BMAL1 gene (see Figures 8(a), 8(b)); and by cotransfecting PPARα/RXRα (see Figure 8(a)) or PPARγ1/RXRα, (see Figure 8(b)) we also found that the PPARα/RXRα or PPARγ1/RXRα genes modulate transcription of mPER indicating that PPARα/RXRα and PPARγ1/RXRα function in the negative regulators of the CLOCK/BMAL1-dependent transcriptions. Recently, Shirai et al. [32] reported that ligands for PPARα and PPARγ do not significantly affect the transcriptional activity of the mouse PER gene. In our study, transcriptional activation of the mPER gene by the CLOCK/BMAL1 gene was altered by transfection of the PPARα/RXRα and PPARγ/RXRα genes compared with that of the control, in apparent contradistinction to the results of Shirai et al. However, we used charcoal-treated medium, which would not contain intrinsic ligands for PPARα, PPARγ, and RXRα making the difference between the two sets of results difficult to interpret.

We believe that the change in transcriptional activity of AOX, CRBPII, and HMG-CoA synthase caused by the presence of the CLOCK/BMAL1 heterodimer may be regulated via the PPRE ($5^{\prime }$-AGGTCA-$3^{\prime }$). The PPRE is the site of action not only of PPARα/RXRα but also of PPARβ/RXRα and PPARδ/RXRα.

In conclusion, the results of this study suggest that there is crosstalk between binding to the PPRE and binding to the E-box by the *CLOCK/BMAL1* gene and by the *PPARα/RXRα* or *PPARγ/RXRα* genes, and that modulation of the PPRE by the *CLOCK/BMAL1* gene affects the activity of the *PPARα/RXRα* gene, the *PPARγ/RXRα* gene, and/or PPARα/γ ligand/activators.

## Figures and Tables

**Figure 1:**
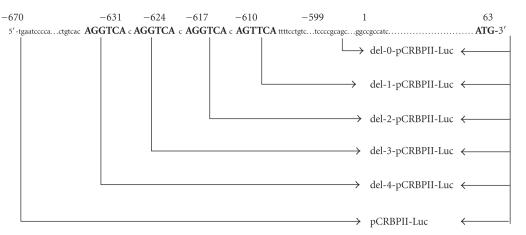
Delations of PPAR response elements ($5^{\prime }$-AG(G/T)TCA-$3^{\prime }$) from the promoter region of the cellular retinol binding protein II (CRBPII)-Luc plasmid.
